# Use of the Brief Suicide Cognitions Scale as a screening tool to identify latent risk in a gambling clinic sample

**DOI:** 10.3389/fpsyt.2026.1736329

**Published:** 2026-02-23

**Authors:** M. David Rudd, Oliver Kratholm, James P. Whelan, Meredith K. Ginley, Andrea Pérez-Muñoz, Vivian L. Gleason, Burak Tuna, Jacob Tempchin

**Affiliations:** 1 Department of Psychology, University of Memphis, Memphis, TN, United States; 2 Department of Psychology, University of Memphis and the Tennessee Institute for Gambling Education and Research, Memphis, TN, United States; 3 Department of Psychology, East Tennessee State University and the Tennessee Institute for Gambling Education and Research, Johnson, TN, United States

**Keywords:** Brief Suicide Cognitions Scale, latent suicide risk, screening, treatment add-ons, treatment-seeking for gambling problems

## Abstract

**Introduction:**

The current study explored the clinical utility of the Brief Suicide Cognitions Scale Q10 (B-SCS) in a sample of individuals seeking treatment for gambling problems but not experiencing an acute suicidal crisis (N = 80).

**Methods:**

The sample specific B-SCS cut score was determined using a normative sample comparison and its subsequent ability to identify latent suicide risk across a range of established risk factors was explored.

**Results:**

Findings suggests that those scoring above the B-SCS cutting score possess elevated latent suicide risk that can be both strategically identified and targeted despite the absence of an acute suicidal crisis.

**Discussion:**

These findings underscore the notion that individuals seeking treatment for gambling disorder exhibit elevated baseline vulnerabilities consistent with those observed in acutely suicidal populations, reinforcing the importance of comprehensive suicide risk assessment and early intervention with this population. Clinical implications of the findings and study limitations are discussed.

## Introduction

The link between problem gambling and elevated suicide risk has been demonstrated across the full spectrum of suicidality, including suicidal ideation (1, 2), suicide attempts (3, 4), and death by suicide (5). A summary statistic presented by a recent systematic review reported that individuals seeking treatment for and/or gambling-related clinical populations had a 22 to 81 percent chance of experiencing suicidal ideation and 7 to 30 percent chance of having made a suicide attempt (6). Despite recognition of elevated suicide risk among problem gamblers, particularly those presenting for treatment who will need providers to be able to accurately assess and skillfully assist them with mitigating risk, studies examining the usage and efficacy of suicide assessment and screening tools with treatment-seeking problem gamblers remains exceedingly scarce. Consequently, there is a need to clarify best practices for suicide risk assessment to guide screening in this population.

Those seeking treatment for gambling disorder often only seek treatment after experiencing significant individual and interpersonal losses (7). Feelings of indebtedness and shame occur at high levels in individuals with gambling disorder (6) and may connect gambling-related symptoms to suicidality via the interpersonal theory of suicide (8). The psychological impact of gambling disorder can reach well beyond shame, guilt, and self-blame with a significant toll on identity and self-efficacy (9), consistent with the identity and agency themes measured by the Brief Suicide Cognitions Scale (B-SCS) (10). Accurately identifying the need for suicide-specific interventions in a population demonstrated to have heightened vulnerability to suicide, but not necessarily in acute suicidal crisis, would allow an opportunity to both identify and address underlying vulnerability directly from a proactive and preventive perspective prior to the emergence of an acute suicidal crisis.

The limited research focused on suicide-related assessment in treatment-seeking gamblers has primarily used the Patient Health Questionnaire 9 (PHQ-9; 11) to screen for suicide risk (12). This approach follows recommendations from the National Council on Problem Gambling in the *Gambling Assessment Manual* (13), which identifies the PHQ-9 and the Ask Suicide Screening Questions (ASQ; 14) as suggested measures for assessing mental health and suicide risk in patients identified as having an increased risk for problem gambling. However, these measures are likely suboptimal for detecting suicide risk among gamblers, particularly when used as the primary means of determining risk. Namely, the PHQ-9 is an instrument designed to measure depression and includes only a single screening item for suicidal ideation which can result in high levels of misclassification (15). The item content more accurately reflects morbid thoughts and self-harm rather than suicidality. When used alone, the PHQ-9 routinely fails to identify suicide risk accurately (16). Similarly, the ASQ contains only four dichotomized (yes/no) questions, limiting its ability to meaningfully capture varying degrees of intensity in experiences or comprehensive risk factors consistent with latent suicide risk.

A strategic and sensitive method of assessment for suicide risk screening may be particularly important in treatment-seeking gamblers, given the unique challenges associated with help-seeking in this population. While many who seek treatment are not experiencing an acute suicidal crisis at intake, non-disclosure of high-risk behaviors is not uncommon for problem gamblers who are experiencing shame and stigma around their symptoms, making suicide risk assessment particularly challenging (7, 17). Additionally, the same significant gambling-related financial losses that lead to treatment-seeking, such as a need to file for bankruptcy, can lead to a state of hopelessness and may be independently and additively related to suicide risk (18).

Potential clients who gamble may also underreport certain behaviors and outcomes, such as betting frequency and monetary losses when completing self-report measures (19). Similar patterns of underreporting have been observed among non-gambling populations who later attempted or died by suicide (20). In a study examining social desirability bias among those who gamble, Kuentzel et al. (21) found that participants were more likely to respond in socially desirable ways (underreporting or not reporting behaviors assumed to be perceived as negative), especially when responding to measures that explicitly tapped gambling-related problems, compared to measures that quantitatively measured gambling-related behaviors. The authors suggest that those who gamble may find it more difficult to acknowledge or report the specifics of their behaviors than report facts on the quantity of gambling-related losses. When considering these findings together, it is reasonable to assume that similar reporting patterns are present among gamblers completing suicide screening measures. Given that traditional approaches to suicide risk assessment demonstrate limited predictive value (22), tendencies for underreporting and non-disclosure among those seeking treatment for gambling problems may further limit the clinical utility of traditional measures like the ASQ or the Columbia Suicide Severity Rating Scale (C-SSRS; 23), both of which rely on direct and specific questions about suicidal thinking and behavior. Identifying instruments that overcome these unique challenges may enable researchers and clinicians to better recognize and respond to latent suicide risk in this population, particularly among those not presenting in an acute suicidal crisis when seeking treatment for problem gambling.

The B-SCS (24) has the potential to help navigate some of these challenges and overcome the limitations of traditional suicide screening tools in this clinical setting. The B-SCS taps suicide cognitions related to motivation to die without using explicit suicide specific language, a feature unique to the B-SCS, which may be particularly advantageous for assessing latent suicide risk among gambling populations. In the context of suicide risk assessment, some gamblers may find it easier to honestly and accurately respond to screening questions when they are not explicitly asked about suicidal thoughts and/or behaviors that may be perceived as negative or socially unacceptable. Regardless, the B-SCS should ideally be used *in addition* to traditional screening tools that ask specifically about suicidal thoughts and behaviors, as it taps unique elements of suicide risk. Even among non-gambling samples, the stigma of suicidal thinking may contribute to the underreporting of ideation or attempts on screening tools (25). Rather than directly asking respondents about suicidal ideation, the B-SCS assesses the core beliefs of unlovability (i.e., that one is unlovable/a burden/defective/a failure), unsolvability (that one’s problems are unsolvable), and unbearability (that one’s feelings are unbearable) which are considered central to latent suicide risk and to heightened vulnerability to discrete suicidal crises (26).

Exploring the B-SCS’s ability to identify latent suicide risk in a sample of those seeking treatment for gambling problems not experiencing an acute suicidal crisis is necessary to determine the clinical utility of this measure with this high-risk population. Recent work established a cutting score of nine for the B-SCS that was uniform across multiple clinical samples at elevated risk for suicide, including those admitted to an inpatient unit secondary to suicidality, those presenting in an emergency department in acute suicidal crisis, and those seeking outpatient care secondary to suicidal ideation and/or a suicide attempt (see 10 for the findings across clinical samples and study methodology). The current study sought to confirm the B-SCS cut score with a gambling disorder treatment-seeking sample by comparing item endorsement rates across multiple clinical samples (i.e., those admitted to an inpatient unit secondary to elevated suicidality and those presenting in an emergency department in acute suicidal crisis) and a normative population (a college student sample). Additionally, we explored the presence of latent suicide risk by comparing indicators of elevated suicide risk for those scoring above and below the cut score in a sample of treatment-seeking problem gamblers not experiencing an acute suicidal crisis (including scores on the AUDIT-C, INQ, and GBQ as detailed below). For clarification, the term “latent suicide risk” is used to characterize elevated vulnerability rather than the idea of prospective prediction of suicidality. Above and below B-SCS cutting score comparisons were limited to those measures used in the gambling clinic routine screening and assessment procedures.

## Methods

### Participants

Participants included individuals presenting for treatment for gambling disorder at a United States-based outpatient psychological treatment center that specializes in helping those with gambling problems. This study focused on the subset of clinic clients who completed suicide-related measures as part of the intake process between March 2024 and December 2024, resulting in a sample size of 80. The sample includes all those completing screening between the dates noted, with no participants excluded secondary to a suicidal crisis. The average age of the sample was 44 years (range from 23 to 72 years of age). In terms of gender, 71% self-reported as male, 25% as female, and 4% preferred not to report. With respect to ethnicity, 76% self-reported as White, 16% Black, 1% Hispanic, 1% Native American, and 6% as multi-racial. Forty-five percent of the sample were married, 36% never married, 5% separated, and 14% divorced. Seventy percent were employed full-time, 13% retired, 8% employed part-time, 8% not employed, and 1% reported to be full-time students. As for education, 35% reported have a bachelor’s degrees, 14% an associate’s degree, 16% a master’s or doctoral degree, 25% reported some college, 9% a high school or GED degree, and 1% some high school experience. Regarding gambling behaviors, 96% reported gambling within the past month and 90% reported financial losses within the past month, ranging from $10 to $80,000, with a mean of $8,032 and a median of $4,000, with 20% reporting bankruptcy. The mean age of their first gambling experience was 26 years (SD = 24.6; range of 10–55). A total of 28 (35%) reported engaging in unspecified illegal activity related to gambling.

### Study measures

#### Demographic information

Demographic information was obtained from self-report forms completed by individuals presenting for outpatient gambling treatment prior to the initial clinical intake. Variables included information used to better describe the sample: age, gender, race, ethnicity, sexual orientation, marital status, employment status, highest level of completed education, and military status.

#### Brief Suicide Cognitions Scale

The original Suicide Cognitions Scale (SCS) is an 18-item measure designed to capture core beliefs related to motivation to die (e.g., unlovability, unbearability, unsolovability) that are associated with suicide risk, including suicide ideation and suicide behaviors (27). The measure has undergone multiple revisions and modifications, all with careful evaluation of scale psychometrics and factor structure (e.g., SCS-R 28; BSCS-6 item, 24). For the purpose of this study, a 5-item version of this measure that excludes an item mentioning “suicide” directly in the item content was be used. Items are rated on a 5-point Likert scale ranging from 1 (disagree strongly) to 5 (agree strongly). Total scores on this abbreviated measure range from 5 to 25, with higher scores indicating greater suicide-related beliefs. The B-SCS demonstrates good predictive validity for suicide attempts and suicide ideation severity, as well as good internal consistency, reliability, and predictive validity for identifying individuals at greater risk of suicidal behaviors, coupled with a unidimensional factor structure (24). Differential performance and related psychometric properties of the 5-item vs. 6-item versions of the B-SCS are presented in detail elsewhere (10), but it is important to note the 5-item scale has retained excellent psychometric properties, including a unidimensional factor structure. The coefficient alpha for the current study was.89.

#### Interpersonal needs questionnaire

The Interpersonal Needs Questionnaire (INQ; 29) is a 15-item self-report measure designed to capture factors related to the interpersonal theory of suicide (30). This study used a 9-item version of the INQ that included items measuring acquired capability for suicide across two central factors, including perceived burdensomeness (e.g., “These days, I think I am a burden on society” or “The people in my life would be happier without me”) and thwarted belongingness (e.g., “These days, I feel disconnected from other people” or “I feel like I belong”) Items are rated on a 5-point Likert scale ranging from 0 (Not at all like me) to 4 (Very much like me). The coefficient alpha for the current study was.92.

#### Patient Health Questionnaire

The PHQ-9 is a 9-item self-report measure assessing for the presence and severity of depression over the past two weeks (11). Items are measured on a 4-point Likert scale ranging from 0 (Not at all) to 3 (Nearly every day). Total scores range from 0–27 and typically include the following severity ranges: none/minimal (0–4), mild (5–9), moderate (10–14), moderately severe (15–19), and severe (20–27). The PHQ-9 has demonstrated good sensitivity, specificity (88%), and has been validated as a measure of depression severity (11). The coefficient alpha for the current study was.93.

#### Wish to live and wish to die

Wish to Live (*I wish to live to the following extent*) and Wish to Die (*I wish to die to the following extent*) was measured on a 9-point Likert scale ranging from 0 (*not at all*) to 8 (*very much*). Higher scores indicate either a greater wish to live or a greater wish to die, respectively.

#### Depression severity

Depression severity was measured on a 5-point Likert scale ranging from 0 (*not at all*) to 5 (*severe).* Higher scores indicate more severe self-rated depression.

#### Alcohol disorders identification test

The AUDIT-C is a 3-item alcohol screening measure typically used to identify individuals endorsing symptoms of alcohol use disorder (31). Items are rated on a 5-point scale measuring frequency (e.g., 0, never to 4, four or more times per week). Total scores range from 0 (indicative of individuals that do not drink) to 12 (indicative of risky drinking behavior). A score of 5 or greater was considered a positive alcohol screen. The coefficient alpha for the current study was.83.

#### Gambler’s Belief Questionnaire

The Gambler’s Belief Questionnaire (GBQ) is a 20-item self-report measure utilized to assess cognitive distortions surrounding an individual’s gambling behavior (e.g., I have a “lucky” technique that I use when I gamble; (32). The GBQ has been validated, maintained good internal consistency (α=.92) and adequate test-retest reliability (*r* =.77; 32). The questionnaire is anchored on a 7-point Likert-type scale from 1 (Strongly Agree) to 7 (Strongly Disagree) with a possible range of 1 to 140. All items were reverse-scored so that higher scores were indicative of greater gambling-related cognitive distortions. Higher scores (i.e., 70 >) on the GBQ are associated with individuals who meet the criteria for gambling disorder (32). The coefficient alpha for the current study was.92.

### Analysis

Data were analyzed using IBM SPSS Version 29 (IBM-Analytics, New York, USA). We calculated the B-SCS cutting score following the recommendations of Jacobson and Truax (33), using a normative comparison sample from one of our previous studies (10). Considering the limitations of a small clinical sample not presenting in acute suicidal crisis, we subsequently examined the presence of latent suicide risk in three stages. First, we compared the rates from those who screened positive on the PHQ-9 and compared to participants who screened positive on the B-SCS, using the identified cutting score for problem gamblers. Second, we reviewed B-SCS item endorsement rates compared to a normative sample and clinical samples known to be at elevated risk for suicide (see 10 for a complete description of these samples and corresponding methodology). Finally, we compared those that scored above and below the B-SCS cutting score across a range of frequently used measures indicating elevated suicide risk including the PHQ-9, wish to live/die ratings, depression severity rating, hopelessness rating, and the INQ subscales. We also explored mean comparisons on the GBQ and overall gambling severity. Given the prominence of identity-related disturbance, shame, and guilt common in individuals struggling with a gambling disorder (e.g., 9) and the potential for elevated suicide risk (1–5), it is important to explore and identify potential indicators of latent suicide risk in clinical samples not experiencing an acute suicidal crisis.

Although findings and observed differences are consistent with our original hypotheses, given our familiarity with the clinical phenomena studied, the analysis was *post-hoc* in nature and the analytic plans were not preregistered.

## Results

Clinically significant change (CSC) was calculated using the methods recommended by Jacobson and Truax (33), with values indicating appropriate cutting scores for differentiating “functional versus dysfunctional” B-SCS scores. When the distributions of scale scores between groups with and without a condition of interest overlap, Jacobson and Truax recommend defining CSC as the midpoint between the mean scores of the two groups. In this analysis, the gambling treatment-seeking sample served as the group with the condition of interest and the normative student sample from our previously published work (in which participants in acute suicidal crisis were excluded) served as the normative group without the condition of interest (10).

In accordance with the recommendation of Jacobson & Truax (33), we calculated clinically significant change (CSC) as the midpoint between the mean normative sample (*M* = 6.31, *SD* = 2.85) and the gambling treatment-seeking sample (*M* = 12.0, *SD* = 4.58). Consistent with other clinical samples, the identified cutting score was 8.67, rounded up to nine, and was consistent with our previous work with inpatient, outpatient, and emergency department samples all experiencing acute suicidal crises (10), despite this sample of treatment-seeking problem gamblers not experiencing acute suicidal crisis. The identified cutting score for the other clinical samples were all nine.

Responses to the PHQ-9, item 9, revealed 71% (n=57) denied any “thoughts that you would be better off dead or of hurting yourself in some way” while 29% (n=23) screened positive by endorsing thoughts from “several days” (19%), to “more than half of the days” (6%) to “nearly every day” (4%). Of those that screened positive on the PHQ-9 (n=23), 100% scored above the B-SCS cutoff, with a mean score for this subsample of 15 (SD = 4.66). In contrast to the PHQ-9 with 29% screening positive for suicide risk, the B-SCS identified a total of 61 (76%) of the sample screening positive, suggesting evidence of latent suicide risk and consistent with prior findings indicting higher rates of suicide risk among problem gamblers (6). Additionally, the BSCS cutting score correctly captured 39 out of 48 with past history of ideation (81%) and 9 out of 13 with past history of suicide attempts (69%), compared to the PHQ-9 item 9 screening captured only 21 out of 48 (44%) of those with both with a past history of suicidal ideation and those with previous attempts.

**Figure 1:**
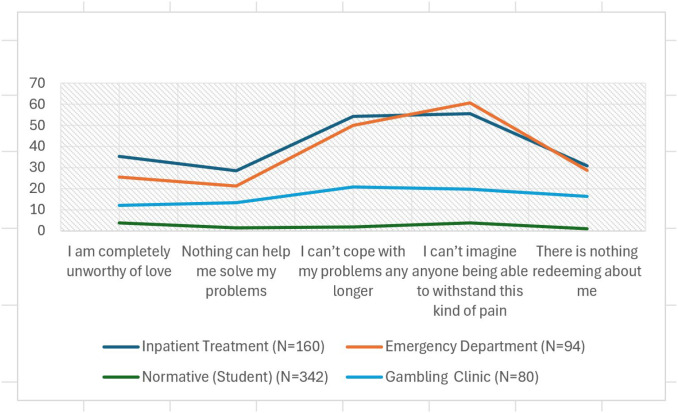
Endorsement rates across samples.

**Table 1** provides a detailed comparison B-SCS item endorsement rates for each item across inpatient, emergency department, normative, and the gambling clinic sample. **Figure 1** provides a graphic illustration of these differences. While item endorsement rates in the gambling treatment-seeking sample were lower than those observed in clinical samples experiencing acute suicidal crises, they were distinct from the normative comparison sample, indicating meaningful differences. The differences were further examined by comparing participants scoring above and below the B-SCS cutting score. As evidence of elevated latent suicide risk among those screening positive on the B-SCS, **Table 2** includes mean comparisons for those scoring above and below the cutting score across multiple measures available for this sample. As **Table 2** indicates those screening positive scored significantly higher on the PHQ-9 total score, overall depression severity, wish to die, gambling severity, and perceived burdensomeness, all of which are established risk factors with a wealth of support (34). Additionally, those screening positive on the B-SCS scored significantly lower on wish to live, further supporting the utility of the B-SCS in identifying individuals with heightened latent suicide risk. Interestingly, scores on the GBQ were similar for those above and below the B-SCS and suggests that gambling specific beliefs may not be helpful in differentiating latent suicide risk.

**Table 1:** B-SCS Item-endorsement rates.

B-SCS items	Inpatient treatment (N = 160)	Emergency department (N = 94)	Normative (student) (N = 342)	Gambling clinic (N = 80)
I am completely unworthy of love	35.3	25.6	3.7	12.0
Nothing can help me solve my problems	28.4	21.2	1.5	13.4
I can’t cope with my problems any longer	54.2	50.0	1.9	20.9
I can’t imagine anyone being able to withstand this kind of pain	55.5	60.6	3.7	19.7
There is nothing redeeming about me	30.9	28.7	1.1	16.3

Item endorsement on the B-SCS items is represented by any level of agreement with an item (i.e., a score of 4 or 5 on the 1–5 scale representing agree or strongly agree).

**Table 2:** Mean comparisons above and below B-SCS cutoff score.

Scale	B-SCS cutoff above mean (n = 60)	B-SCS cutoff below mean (n = 18)	SD (above/below)	F-value	Sig. (p<.05)
PHQ-9	13.38	6.94	3.93/6.25	8.34	.005
Depression-Severity	3.12	2.06	0.87/1.23	8.98	.004
Wish to Die	1.14	.32	0.67/1.79	7.33	.008
Wish to Live	6.88	7.44	0.65/1.62	14.14	<.001
INQ-Capability	1.90	2.06	0.95/1.01	0.02	NS
INQ-Belongingness	2.60	1.42	0.96/0.94	0.02	NS
INQ-Burdensomeness	1.77	.53	0.63/1.22	6.72	.011
INQ-Hopelessness	1.63	.82	1.06/1.11	.318	NS
AUDIT-C	2.51	2.83	2.77/2.67	0.40	NS
Gambling Severity	2.39	2.16	0.89/0.64	8.24	.005
GBQ	82.3	65.7	20.14/17.06	0.71	NS

NS, not significant.

Significance levels adjusted for multiple comparisons using a Bonferroni Correction.

## Discussion

The current results offer several clinically relevant findings in a sample of individuals seeking treatment for problem gambling but not experiencing an acute suicidal crisis at the time of intake. First, despite not being in acute suicidal crisis, the calculated B-SCS cutting score was comparable to clinical samples known to be at significantly elevated suicide risk, including those admitted to an inpatient facility specifically for elevated suicide risk and those in an emergency department secondary to acute suicidal thinking and/or suicidal behavior. This finding suggests that similar latent risk elements may be present among treatment-seeking gamblers. To clarify, the term “latent suicide risk” is used to characterize elevated vulnerability rather than the idea of prospective prediction of suicidality. Exploring the issue of predictive validity is not possible in the current study. Second, item-endorsement rates for those seeking treatment for gambling problems were noticeably higher than a normative comparison group and, although lower than the clinical samples mentioned, clearly suggestive of potential for latent suicide risk consistent with fluid vulnerability theory (26). Subsequent mean comparisons across a range of measures demonstrated to indicate elevated suicide risk (34) suggests that those scoring above the B-SCS cutting score possess elevated latent suicide risk when compared those below the cutting score and reflected in the core themes of suicidogenic beliefs of unlovability, unbearability, and unsolvability. These findings underscore the notion that individuals seeking treatment for gambling disorder exhibit elevated baseline vulnerabilities consistent with those observed in acutely suicidal populations, reinforcing the importance of comprehensive suicide risk assessment and early intervention with this population.

Fluid vulnerability theory (FVT; 26) posits that suicide risk fluctuates over very brief periods of time, with discrete episodes of risk and variable thresholds for risk activation of future episodes across individuals depending on developmental, psychological, emotional and situational factors (e.g., 35). Ecological momentary assessment findings over the course over the past decade support this model, demonstrating discrete, time-limited episodes of suicide risk and varying thresholds of vulnerability for future episodes consistent with FVT (e.g., 36). Identifying latent risk in populations like treatment-seeking problem gamblers offers an opportunity to intervene from a clinically strategic, preventive, and proactive perspective concerning elevated suicide risk. Additionally, the B-SCS identifies core themes of suicidogenic beliefs related to individual vulnerability revolving around core identity (i.e., unlovability) and agency (unsolvability and unbearability) themes, both of which have clinical relevance for individual vulnerability that extends well beyond acute suicidal crises and may also be related to gambling behaviors and related decision-making. Beyond individual predispositions, the additive harms of gambling disorder – often particularly acute in populations seeking treatment – may heighten suicide risk through intensified hopelessness, shame, perceived burdensomeness, financial distress, and social withdrawal.

When clinicians recognize the need for suicide-specific interventions secondary to identifying latent risk, it is important to use a suicide-specific treatment add-on that has empirical support and allows clinicians to tailor treatment to the individual regardless of the original presentation and central focus of clinical care (e.g., in this case problem gambling). One simple and easy-to-implement suicide-specific treatment add-on option is described elsewhere is a modification of brief cognitive behavioral therapy for suicide prevention (BCBT-SP) (37) which may work well for this particular population. The specific elements of the treatment add-on include narrative suicide risk assessment and conceptualization, crisis response planning, lethal means counseling, limited emotion regulation strategies, and a relapse prevention plan) – all of which can be delivered in 4.5 hours (a 63% reduction from the full BCBT-SP protocol) (38, 39) and offer a time-limited compliment to treatment for a primary presenting concern such as gambling disorder.

Although current findings offer some important insight into the nature of latent suicide risk in those seeking treatment for gambling disorder and can inform preventive clinical implications, there are limitations of the current project that warrant mention. First, the sample is small. Although this sample produced cutoff scores comparable with other clinical samples, further work is needed to determine if current findings generalize to larger samples of treatment-seeking gamblers. Second, while more comprehensive than the PHQ-9, a limited number of suicide-specific risk measures were utilized, limiting the ability to fully recognize the nature and severity of acute suicidality in the sample. Third, the study is cross-sectional, with no follow-up to explore predictive validity and vulnerability to future episodes of elevated acute suicidality. Further, as a specialty clinic focused on the treatment of gambling problems, comorbid conditions were not systematically assessed in the intake packet, but over the course of the case conceptualization and with consideration of co-occurring treatment or stabilization for these disorders clients may be receiving in other settings. While this process informed a client-centered assessment approach prioritized by the clinic where data was collected, it limits the understanding of how co-occurring disorders-such as substance use disorders and mood disorders-may have influenced suicide risk within this population. Fourth, there is conceptual overlap between the B-SCS and other measures routinely used in studies targeting suicide risk (e.g., measures of hopelessness, burdensomeness, capability to die). Finally, it is also important for future work to explore the clinical utility of suicide-specific treatment add-ons with groups like those experiencing gambling problems. It is important to acknowledge, though, that current findings address only the issue of latent risk and discussion of suicide-specific treatment add-ons is something we will explore empirically in future studies with this population. Despite these limitations, current findings offer preliminary evidence for assessment strategies to identify latent risk in problem gamblers not in acute suicidal crisis that can be the basis for preventive individualized treatment to address variables linked to underlying individual risk (e.g., unlovability, unbearability, and unsolvability).

Given the hesitation to seek treatment for many of those experiencing gambling-related problems (40), current data suggests the B-SCS might have unique potential as a screener for identifying latent risk on publicly available websites addressing gambling-related problems or offering support and/or treatment services for those with gambling-related problems. If used outside of a clinical context, however, it is recommended that the scale not be identified as “screening” for latent suicide risk. Rather, it can be introduced as a scale that helps identify feelings about oneself and self-efficacy that might co-occur when someone is experiencing gambling problems and that it might also prove helpful to discuss with a clinician and/or a therapist. Similarly, given the nature of the B-SCS items, it is important to always link and highlight support and treatment services if the B-SCS is utilized in non-clinical settings.

## Data Availability

The raw data supporting the conclusions of this article will be made available by the authors, without undue reservation.
